# PPARγ S273 Phosphorylation Modifies the Dynamics of Coregulator Proteins Recruitment

**DOI:** 10.3389/fendo.2020.561256

**Published:** 2020-11-27

**Authors:** Marieli Mariano Gonçalves Dias, Fernanda Aparecida Heleno Batista, Thais Helena Tittanegro, André Gustavo de Oliveira, Albane Le Maire, Felipe Rafael Torres, Helder Veras Ribeiro Filho, Leonardo Reis Silveira, Ana Carolina Migliorini Figueira

**Affiliations:** ^1^Brazilian Biosciences National Laboratory (LNBio), Brazilian Center for Research in Energy and Materials (CNPEM), Campinas, Brazil; ^2^Graduate Program in Functional and Molecular Biology, Institute of Biology, State University of Campinas (Unicamp), Campinas, Brazil; ^3^Mitochondrial Molecular Biology Laboratory, Obesity and Comorbidities Research Center (OCRC), Campinas, Brazil; ^4^Department of Structural and Functional Biology, Institute of Biology, University of Campinas (UNICAMP), Campinas, Brazil; ^5^Centre de Biochimie Structurale CNRS, Université de Montpellier, Montpellier, France

**Keywords:** PPARgamma, coregulator interaction, Ser273 phosphorylation, insulin resistance, coactivator, corepressor, nuclear receptors

## Abstract

The nuclear receptor PPARγ is essential to maintain whole-body glucose homeostasis and insulin sensitivity, acting as a master regulator of adipogenesis, lipid, and glucose metabolism. Its activation through natural or synthetic ligands induces the recruitment of coactivators, leading to transcription of target genes such as cytokines and hormones. More recently, post translational modifications, such as PPARγ phosphorylation at Ser273 by CDK5 in adipose tissue, have been linked to insulin resistance trough the dysregulation of expression of a specific subset of genes. Here, we investigate how this phosphorylation may disturb the interaction between PPARγ and some coregulator proteins as a new mechanism that may leads to insulin resistance. Through cellular and *in vitro* assays, we show that PPARγ phosphorylation inhibition increased the activation of the receptor, therefore the increased recruitment of PGC1-α and TIF2 coactivators, whilst decreases the interaction with SMRT and NCoR corepressors. Moreover, our results show a shift in the coregulators interaction domains preferences, suggesting additional interaction interfaces formed between the phosphorylated PPARγ and some coregulator proteins. Also, we observed that the CDK5 presence disturb the PPARγ-coregulator’s synergy, decreasing interaction with PGC1-α, TIF2, and NCoR, but increasing coupling of SMRT. Finally, we conclude that the insulin resistance provoked by PPARγ phosphorylation is linked to a differential coregulators recruitment, which may promote dysregulation in gene expression.

## Introduction

Peroxisome proliferator-activated receptor gamma (PPARγ) is closely linked to energy homeostasis regulation, playing important role in adipogenesis, lipid and carbohydrates metabolism, insulin sensitivity, cell proliferation, and inflammatory processes. This nuclear receptor (NR) acts as a metabolic sensor of dietary lipids and is a crucial metabolism modulator (1, 2), regulating diabetes through cytokines and hormones, such as TNFα and leptin genes (2–4). Like other NR superfamily members, PPARγ is activated by natural ligands, such as fatty acids and their metabolites, and by synthetic ligands such as the insulin sensitizers Thiazolidinediones (TZDs), as Rosiglitazone and Pioglitazone, drugs commonly used in type 2 diabetes treatment.

The canonical transcriptional activity of PPARγ occurs through its interaction with several cofactors, which activate or suppress gene transcription. In the absence of ligands, the inactive conformation of helix 12 (H12) of PPARγ ligand binding domain (LBD), favors the binding of corepressor proteins, such as silencing mediator of retinoid and thyroid hormone receptor (SMRT) and the nuclear receptor corepressor 1 (NCoR). These proteins form a corepressor complex with histone deacetylases (HDAC) repressing target gene transcription (5). In the presence of ligands, the receptor undergoes a conformational change, that reallocates H12, forming a charge clamp between H3 and H12 (6). This conformation leads to corepressors dissociation and coactivators recruitment, forming a coactivator complex by the recruitment of other proteins, as well as histone acetyltransferases (HAT) and other general transcription factors, promoting the transcription of the target gene (7). Beyond this canonical transcriptional activity, PPARγ can also be regulated by post-translational modifications (PTMs), as acetylation, phosphorylation, SUMOylation, and ubiquitination (8, 9). These fine-tuning adjustment is part of the cell tissue-specific modulation (9, 10) and can dramatically alter the receptor function, as well as its binding to coregulators (11). By all these PTMs, the PPARγ phosphorylation is one of the most studied, and may promote different receptor’s behavior, depending on the residue in which it occurs, and on the enzyme that performs the phosphorylation and/or dephosphorylation (12, 13).

Most of PPARγ phosphorylation were described on its N-terminal domain. The phosphorylation of Y78 is regulated by SRC proto-oncogene, nonreceptor tyrosine kinase (c-SRC), and Protein-tyrosine phosphatase 1B (PTP-1B), and affects the inflammatory response and insulin sensitivity (14). The phosphorylation in S112 by Mitogen-Activated Protein Kinases (MAPKs) pathway (12, 13), and by the Cyclin-Dependent Kinase 7 (CDK7) and 9 (CDK9) (15, 16) intensifies the interaction between PPARγ and the circadian clock protein PER2 (Period Circadian Regulator 2) (17), decreasing PPARγ activation trough reduction of both coactivator binding (12) and ligand binding affinity (18). In addition, S133 and T296 residues were also identified as targets to Extracellular Signal-Regulated Kinase (ERK)/Cyclin-Dependent Kinase 5 (CDK5) phosphorylation pathway (19).

Particularly, one special obesity-mediated phosphorylation that targets PPARγ ligand binding domain (LBD) has been associated with insulin resistance (20, 21). This phosphorylation, performed by the CDK5 at PPARγ S273 (or S245 in isoform 1), does not alter the adipogenic activity of PPARγ, but deregulates a subset of genes, that presented altered expression in obesity and diabetes, such as adiponectin and adipsin (20, 21). It is known that this phosphorylation does not changes the occupancy of PPARγ in the chromatin (21), but the mechanism that corelates this phosphorylation to the deregulation of these specific genes is still unknown. Various PPARγ ligands are capable of inhibit this phosphorylation. Among them is the insulin-sensitizer class of drugs TZDs, which owns familiar anti-diabetic actions but presents negative side effects due to its strong agonism. On the other hand, some partial agonists, such as MRL24 (20), SR1664 (21), GQ-16 (22), UHC1 (23), F12016 (24), L312 (25), Chelerythrine (26), and AM-879 (27), have been identified to inhibit this PTM without the agonist activity. Structural data analysis showed that PPARγ ligands that inhibit S273 phosphorylation do not make direct contact with this residue, but induces structural modifications in PPARγ:CDK5 interaction interface. Such ligands fit into binding pocket promoting an interaction network that protects S273, blocking its phosphorylation (28). Therefore, the most recent strategy of PPARγ modulation have been target the partial agonism of receptor, aiming S273 phosphorylation inhibition.

Mastery and manipulation of the mechanisms involved in this phosphorylation pathway can be a promising approach in the improvement of metabolic disorders therapies. Also, it is known that phosphorylation may contribute to increased coactivator and decreased corepressor activity (29). For example, it is reported that the Thyroid Hormone Receptor 3-Associated Protein (THRAP3), directly interacts with PPARγ specifically when S273 is phosphorylated, acting as a specialized coregulator that docks on certain phosphorylated transcription factors (30). Moreover, the corepressor NCoR was reported as an adaptor protein that enhances the ability of CDK5 to associate with and phosphorylate PPARγ (31).

Here, we demonstrate that the dysregulation caused by Ser273 phosphorylation might occur through the differential recruitment of coregulatory proteins, causing differences in the target genes expression. By using five coregulators reported to interact with PPARγ in adipogenesis, the PGC1-α, TRAP220 and TIF2 coactivators, and the SMRT and NCoR corepressors (32–35), we evaluated how the PPARγ S273 phosphorylation modifies its interaction with coregulators. Our results show that both the presence and absence of phosphorylation at S273 can alter PPARγ activation and its binding profile with some coregulators. The absence of phosphorylation can lead to an increased activation of PPARγ due to a higher interaction with coactivators and decreased interaction with corepressors. Additionally, we found that the CDK5 presence also disrupts this coregulator harmony. Finally, we also hypothesize that additional interfaces may be formed in coregulator:PPARγ interaction due to differential PPARγ phosphorylation states.

## Materials and Methods

### Plasmids for Cell Assays

Cell assays were performed using the following plasmids: pBIND-PPARγ harboring a chimeric protein composed of Gal 4 DBD and the PPARγ LBD region (aa 238-503), pGRE-LUC (containing the upstream activating sequence of Gal 4 followed by a firefly luciferase reporter gene), pRL-TL (which constitutively express Renilla reniformis luciferase, used as transfection control for vector normalization). All the coregulators constructs were inserted into the commercial vector pM (Clontech), which contains the Gal 4 DBD. The Gal- PGC1-α (containing mouse PGC1-α from 136 to 340 amino acids) and Gal-TRAP220 (ID1 + ID2 containing human TRAP220 from 404 to 654 amino acids) are plasmids belonging to the Laboratory of Spectroscopy and Calorimetry (LEC, LNBio/CNPEM, Brazil). Gal-TIF2 [harboring three interaction domains (IDs) of human TIF2 from 624 to 869 amino acids], Gal-SMRT (ID1 + ID2, containing human SMRT from 982 to the C terminus), Gal-NCoR (ID1 + ID2 + ID3 containing mouse NCoR from 1629 to the C terminus), and VP16-PPARγ (harboring the chimeric protein of the LBD region of PPARγ with the transactivation domain of the VP16 Human herpes simplex virus 2) were kindly provided by Dra. Albane Le Maire from Centre de Biochimie Structurale (CBS, CNRS, France). The plasmid pCDNA-CDK5 (which encodes the CDK5 and P35 proteins) were kindly provided by Professor Sang K. Park of Pohang University of Science and Technology.

### Mutations

To evaluate whether S273 phosphorylation would change both activation of PPARγ and its interaction with coregulators, we mutated this residue (target of phosphorylation) in order to mimic the phosphorylated serine and the inhibition of phosphorylation. Mutations of pBIND-PPARγ and VP16-PPARγ at S273 to alanine (PPARγ S273A), used as a constitutive dephosphorylation PPARγ form, and to aspartic acid (PPARγ S273D), used to mimic phosphorylation were performed using Quick Solution of QuickChange site-directed mutagenesis kit (Promega) with pFU DNA polymerase (Promega).

This same strategy was applied to generate Gal-PGC1-α, Gal-TIF2, Gal–SMRT, and Gal-NCoR derivatives harboring mutated IDs. In order to inactivate each ID, for coactivators two specific leucine were substituted by alanine, as Gal-PGC1-α domain LKKLL was mutated to LKKAA (residues 142-146, Gal4-PGC1-α ID1m), Gal-TIF2 had the ID1(residues 641-645) changed from LLQLL to LLQAA (Gal-TIF2 ID1m), the ID2 (residues 689-694) changed from LHRLL to LHLAA (Gal-TIF2 ID2m), and the ID3 (residues 744-749) changed from LRYLL to LRYAA (Gal-TIF2 ID3m). For corepressors, the specifics isoleucine were replaced by alanine, as Gal-SMRT had the ID1 (residues 2094-2098) changed from ISEVI to ISEAA (Gal-SMRT ID1m), and the ID2 (residues 2296-2300) changed from LEAII to LEAAA (Gal-SMRT ID2m), and Gal-NCoR had the ID1 (residues 2073-2077) changed from ICQII to ICQAA (Gal-NCoR ID1m), the ID2 (residues 2277-2281) changed from LEDII to LEDAA (Gal-SMRT ID2m), and the ID3 (residues 1932-1937) changed from IDVII to IDVAA (Gal-SMRT ID3m). The used primers are listed in the **Supplementary Material** and all the mutations and constructs were verified by DNA sequencing.

### Reporter Gene Assays

COS-7 and 293T cells were cultured in DMEM (Dulbecco’s Modified Eagle’s Medium) supplemented with 10% Bovine Fetal Serum (FBS), 1% antibiotics (penicillin and streptomycin) and 0.37% sodium bicarbonate and kept in a humid incubator, at 37°C and 5% CO2. Plasmids transfection were performed using 400ng of each plasmid and the JetPEI (Polyplus) transfecting agent in 3:1 ratio. After 24 h of transfection, 1 μM of Rosiglitazone was added to the wells, which was incubated for more 24 h. The cells were lysed and assayed for reporter expression. Luciferase was measured using the Dual-Luciferase^®^ Reporter Assay System kit (Promega). Luminescence reading was performed on the GloMax^®^-Multi Detection System reader. In each case, we normalized results by co-expressed Renilla luciferase signal. We carried out each transfection in triplicate and repeated each assay three to eight times (36).

To measure possible changes in PPARγ activation in different phosphorylation states, transactivation assays were performed on 293T cells with transient transfection of plasmids pBIND-PPARγ, pBIND-PPARγ S273A, pBIND-PPARγ S273D, pGRE-LUC, pRL-TL as transfection control, and pCDNA3-CDK5. To measure the interaction between coregulators and PPARγ, and possible differences due to different receptor phosphorylation states, mammalian two-hybrid assays were performed in Hek293T cells for corepressor assays and COS-7 for coactivators assays. The plasmids used were: VP16-PPARγ, VP16-PPARγ S273A, VP16-PPARγ S273D, Gal-Coregulators (PGC1-α, Gal-TRAP220, Gal-TIF2, Gal-SMRT, Gal-NCoR, and its mutated derivatives), pGRE-LUC, pRL-TL as transfection control, and pCDNA-CDK5.

The luminescence value was corrected by transfection control (luciferase Firefly/Renilla) and the value of each tested condition was divided by the luminescence value of the experimental control to obtain the activation rate. As negative control of transactivation assays empty pCDNA3.1 vector was used. For mammalian two hybrid assays, the luminescence value of each tested condition was divided by the baseline condition of the experiment, which for the corepressors is the corepressor tested without the presence of PPARγ, and for the coactivators it is the empty Gal4 vector to obtain the interaction rate (37, 38). Data analysis was performed with GraphPad Prism, by two-way ANOVA, comparing the groups treated with Rosiglitazone and untreated of each PPARγ derivative by Bonferroni’s test, with values of p < 0.05.

### Adipocyte Differentiation

3T3-L1 cells were cultured in DMEM medium containing 50 units/ml of penicillin and streptomycin, 3.7 mg/L of sodium bicarbonate and 10% (v/v) of neonatal bovine serum in T125 bottles (Sarstedt). Cells were plated in 6-well plates (Corning^®^) at a density of 2.8 × 10^5^ cell/well and cultivate until reaching 100% confluence. To induce differentiation, cells were initially treated for 48 h with a differentiation induction medium (DMEM medium containing 50 units/ml of penicillin and streptomycin, 3.7 mg/L sodium bicarbonate and 10% SFB, 1 μM dexamethasone, 0.5 mM IBMX (3-isobutyl-1-methylxanthine) and 1 ug/ml of insulin). Forty-eight hours later the medium was changed by maintenance medium (containing DMEM containing 50 units/ml of penicillin and streptomycin, 3.7 mg/L of sodium bicarbonate and 10% of SFB and 1 ug/ml of insulin). Maintenance medium was renewed every 48 h for 7 days. The control condition received no treatment, except for the differentiation cocktail. The treated conditions were: Rosiglitazone (1μM), Roscovitine (10μM) and Rosiglitazone (1μM) + Roscovitine (10 μM). These component concentrations were maintained and renewed every 2 days, along with the change of medium. After the 7 days of treatment, Trizol^®^ RNA extraction was performed as previously described in (39), followed by cDNA preparation with High Capacity cDNA Reverse Transcription kit (Applied Biosystems). The cells were also stained with Oil Red O according to described in (40), and their absorbance at 520 nm was measured by a spectrophotometer. Data analysis was performed with GraphPad Prism, by one-way ANOVA, comparing the different treatments, values of p < 0.05.

### qPCR

For the real-time amplification, we used the SYBR^®^ Green PCR Master Mix (ABI) kit with 0.2 to 0.6 μM of primers in a final reaction volume of 12 μl. The amplifications were performed in the 7500 Real-Time PCR System (Applied Biosystems) thermal cycler with the following protocol: 95°C for 10 min, followed by 40 cycles of 95°C for 10 s and 60°C for 1 min (data collection). The specificity of each reaction was tested using dissociation curves with temperature variation from 65°C to 95°C, with an increase of 0.5°C every 15 s, with continuous fluorescence measurement. The amplifications were performed in triplicates, the negative reaction controls with no-template (NTC), were performed at each amplification to ensure the absence of reaction contamination.

The relative normalized expression calculation was determined by the 2-ΔΔCq method (41), which considers a 100% efficiency for the amplifications, confirmed by the primer efficiency test. Tbp and Rpl27 reference genes were used to normalize the reactions. The data were statistically compared using the Kruskal-Wallis test (non-parametric), followed by the Dunn *post hoc* test, using the Prism 5.01 software (GraphPad Software, San Diego, CA, USA).

### Protein Expression and Purification

pET-28a_PPARγLBD (aa207-aa477), pET-28a_PPARγS237D, and pET-28a_PPARγS237A expression and purification was performed as previously described in (27). pET-15_NCoR (aa2059-aa2297) expression was performed in Escherichia coli BL21 (DE3) strain. Cells were growth in Luria-Bertani medium (LB), at 37°C, until OD600nm = 0.8 and were induced with 1mM Isopropyl β-d-1-thiogalactopyranoside (IPTG) and 10μM ZnCl2, at 22°C for 16 h, 200RPM. Then, bacteria were harvested by centrifugation (20 min at 16,000 rcf at, 4°C), and the pellet was resuspended in lysis buffer (20 mM Tris–HCl pH 7.5, 300 mM NaCl, 5% glycerol, 2 mM β-mercaptoethanol, 100 mM PMSF and 1 mg lysozyme). After 1 h at 4°C, the extract was sonicated on ice bath and the soluble fraction was separated by centrifugation at 36,000 rcf, for 1 h at 4°C.

pGEX-2T_SMRT (aa2041-aa2359) expression was performed in modified Escherichia coli BL21(DE3) strain (42). Cells were growth in LB medium, at 37°C, until OD600nm = 0.88 and were induced with 1mM Isopropyl β-d-1-thiogalactopyranoside (IPTG) at 18°C for 16 h, 200RPM. Then, bacteria were harvested by centrifugation (20 min at 16,000 rcf at 4°C), and the pellet was resuspended in lysis buffer (10 mM NaH_2_PO_4_ pH 7.4, 140 mM NaCl, 2.7 mM KCl, 1mM β-mercaptoethanol). After 1 h at 4°C, the extract was sonicated on ice bath and the soluble fraction was separated by centrifugation at36,000 rcf, for 1 h at 4°C. The supernatant was incubated with previously equilibrated Glutathione Sepharose 4B GST-tagged resin (GR Healthcare) for 3 h. After that, resin solution was transferred to a plastic column and flow through was collected. The resin was washed with (10 mM NaH_2_PO_4_ pH 7.4, 140 mM NaCl, 2.7 mM KCl, 1 mM β-mercaptoethanol) and fractions were eluted with elution buffer (60mM Tris pH 8.0, 10mM reduced glutathione, 1 mM β-mercaptoethanol).

The coactivators pET-28a_PGC1-α (aa138-aa341) and pGEX-2T_TIF2 (aa563-aa757), were expressed in in Escherichia coli BL21 (DE3) strain. Cells were growth in Luria-Bertani medium (LB), at 22°C, until OD600 nm = 0.8 and were induced with 1 mM Isopropyl β-d-1-thiogalactopyranoside (IPTG) for 16 h at 200RPM. Then, bacteria were harvested by centrifugation (20 min at 16,000 rcf at, 4°C), and the pellet was resuspended in lysis buffer (PGC1-α: 20 mM Hepes pH 7.4; 1 M NaCl; 2 mM β-mercaptoethanol; 80 ug lysozyme; 1 mM PMSF. TIF2: 20mM Hepes pH 8; 300 mM NaCl; 5% glycerol; 80 ug lysozyme; 1 mM PMSF). After 1 h at 4°C, the extract was sonicated on ice bath and the soluble fraction was separated by centrifugation at 36,000 rcf, for 1 h at 4°C. PGC1-α affinity purification was performed in TALON^®^ Superflow™ histidine-tagged protein purification resin, the extract was incubated for 2 h, then eluted wit elution buffer (10 mM Tris-Cl pH 8; 10 mM reduced glutathione; 100 mM NaCl). TIF2 affinity purification was performed in previously equilibrated Glutathione Sepharose 4B GST-tagged resin (GR Healthcare) incubated for 16 h, then eluted in elution buffer (200 mM hepes pH 8; 300 mM NaCl; 5% glycerol; 10 mM reduced glutathione; 1 mM DTT). For our purpose, these two proteins did not undergo gel filtration purification.

### Fluorescence Anisotropy Assay

Affinity purified coregulators were labelled with FITC (fluorescein isothiocyanate), in a proportion of 500 ul of coregulator/control affinity elution with 50 ul of 20mM FITC at 4°C for 3 h. The probe excess was removed by a desalting column (HiTrap, 5 ml, GE). To evaluate the affinities between coregulators and PPARγ, serial dilutions of purified PPARγ wild-type (wt) or S273A and S273D mutants (200 μM to 6 nM) were performed using the elution buffer of each coregulator (see Protein Expression and Purification section), in three replicates, in black 384-wellplates (Greiner). The coactivator conditions were incubated also with Rosiglitazone (3× molar excess). In order to measure any unspecific interaction, we performed the same experiment with control expressions of non-induced protein extracts. These extracts were incubated in GST and cobalt resins, labeled with FITC in the same proportion (50 ul in 500 ul of extract elution) and the affinity with PPARγ we and mutants were measured. For each fluorescence curve, the mixtures were submitted to fluorescence anisotropy measurements using ClarioStar^®^ plate reader (BMG) (emission of 520 nm and excitation of 495 nm). Data were analyzed using the software OriginPro 8.6 and Kd were obtained from fluorescence data fitted to binding curves using Hill model.

### Pull-Down

To confirm PPARγ:Coregulators interaction, extracts of 2L of His tagged PGC1-α (aa138-aa341) and NCoR(aa2059-aa2297) protein expression were incubated with 300 ul of TALON^®^ Superflow™ histidine-tagged protein purification resin (GE Healthcare) for 2 h in agitation. The same amount of GST-tagged TIF2 (aa563-aa757) and SMRT (aa2041-aa2359) extracts were incubated with 300 ul of Glutathione Sepharose^®^ High Performance (GE Healthcare) for 16 h in agitation. After initial incubation, resins were washed with 3mL of lysis/wash buffer of each protein (previously described in session 2.6). Then, the resins contained tagged proteins were incubated with purified tag-free PPARγ (aa207-aa477) and PPARγS237A (aa207-aa477) for 2 h at 4°C in agitation. The conditions with the coactivators were added with rosiglitazone which was incubated with PPARγ and PPARγS237A for 20min before being incubated with the resin. After 2 h, the resins were washed with 3mL of lysis wash buffer proper to each coregulator protein. Then, they were eluted in 100 ul of elution buffer of each protein (previously described).

### Western Blotting

To confirm pull-down formed complexes, 50ug of each eluted complex were electrophoresed on 12% polyacrylamide gels and transferred into nitrocellulose membranes (Amersham™ Protran^®^). Membranes were blocked with 3% skim milk in Tris-buffered saline containing 0.1% Tween-20 (T-TBS) for 4h, and then incubated for 16 h at 4°C with the primary antibodies followed by a 2 h incubation with secondary antibodies. Proteins were analyzed using anti-PPARγ (Cell Signaling #2050S), and anti Phospho-CDK Substrate Motif (Cell Signaling #9477).

### In Vitro Phosphorylation Assay

CDK5 mediated phosphorylation of PPARγ and of the PPARγ complexes with PGC1-α, TIF2, SMRT and NCoR from pull down assays were measured by luminescent detection of ADP produced in the *in vitro* phosphorylation reaction, as it was described in (27, 28). We used ADP-Glo™ kinase assay (Promega) following manufacturer’s instructions, in which 15 μM of purified PPARγ LBD and the pull down purified complexes PPARγ + PGC1-α, PPARγ + TIF2, PPARγ + SMRT and PPARγ + NCoR were incubated with 25 ng of purified CDK5/p35, at room temperature for 15 min, in the kinase assay reaction buffer (200mM Tris-HCl, pH 7.4, 100mM MgCl2 and 0.5 mg/ml BSA, SignalChem kinase assay buffer III) added 10 μM of ATP, in 12.5 μl of reaction volume. After the kinase reaction, ADP-Glo™ Reagent was added and the reaction was incubated at room temperature for 40 min. Then, the samples were denaturated at 95°C for 30 s. After this step, the Kinase Detection Reagent was added, and the samples were incubated at room temperature for 30min. Luminescence signal was recorded using GloMax-Multi + Detection System (Promega) microplate luminometer. Statistical analysis was performed with GraphPad Prism, by t-test, with values of p-values < 0.05.

## Results

### The Absence of Phosphorylation Increases PPARγ Activation

**Figure 1:**
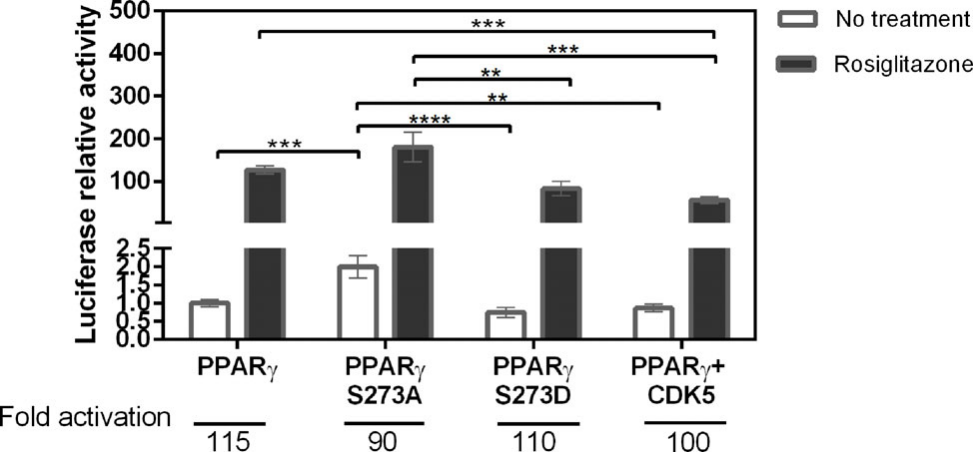
Activation of PPARγ in different phosphorylation states. Transactivation assay with reporter gene in mammalian cells (Hek293T) was used to evaluate the activation profile of PPARγ wt and its mutants in the presence and absence of the Rosiglitazone. The PPARγ S273A mutant prevents the occurrence of phosphorylation, the PPARγ S273D mutant is a structural phosphomimic. The CDK5 enzyme is responsible for the phosphorylation of PPARγ in S273. It is possible to observe that phosphorylation prevention increases activation of PPARγ. Eight assays were performed in biological triplicate with n = 24. Statistical analysis: one-way ANOVA. p ≤ 0.01** p ≤ 0.001***; p ≤ 0.0001****. The phosphorylation inhibitor mutant had greater activation relative to the other conditions.

To measure possible differences in the PPARγ activation due to S273 phosphorylation, we performed gene reporter assay comparing the activation of PPARγ wt, PPARγ S273A, a phosphorylation-defective mutant, and PPARγ S273D, a structural phosphomimic mutant. The lack of phosphorylation of PPARγ S237A and the phosphorylation of PPARγ co-transfected with CDK5 were confirmed by PPARγ immunoprecipitation, followed by western blotting against phosphorylated CDK-5 substrate analysis (**Supplementary Figure 1**). In addition, we measured the PPARγ wt activation in the presence of the CDK5, enzyme responsible for PPARγ S273 phosphorylation. The Rosiglitazone induced PPARγ activation in similar way for both wt and phosphorylated conditions (PPARγ wt, PPARγ S273D, and PPARγ + CDK5), presenting a fold activation of 115, 110, and 100, respectively (**Figure 1**). Interestingly, these results imply that the phosphomimic mutant behaves close to PPARγ wt inside the cells, in the presence and the absence of CDK5 (PPARγ + CDK5), validating the use of this mutant to mimic PPARγ phosphorylation situations. Moreover, PPARγ wt possibly is phosphorylated in this specific cellular assay conditions.

On the other side, PPARγ S273A mutant presented the highest absolute value of Rosiglitazone-induced activation among all the mutants (**Figure 1**); however, its activation fold was the lowest (90-fold). This lower activation ratio reflects the increased basal activation of this mutant (no treatment) that doubled in comparison to PPARγ wt basal activation. These results suggest that the inhibition of S273 PPARγ phosphorylation increases this receptor’s activation, which may be associated to an enhanced dissociation of corepressors and/or to an improvement on coactivators recruitment.

### The Absence of S273 Phosphorylation Increases Both Coactivators Coupling and the Corepressors Dissociation

**Figure 2:**
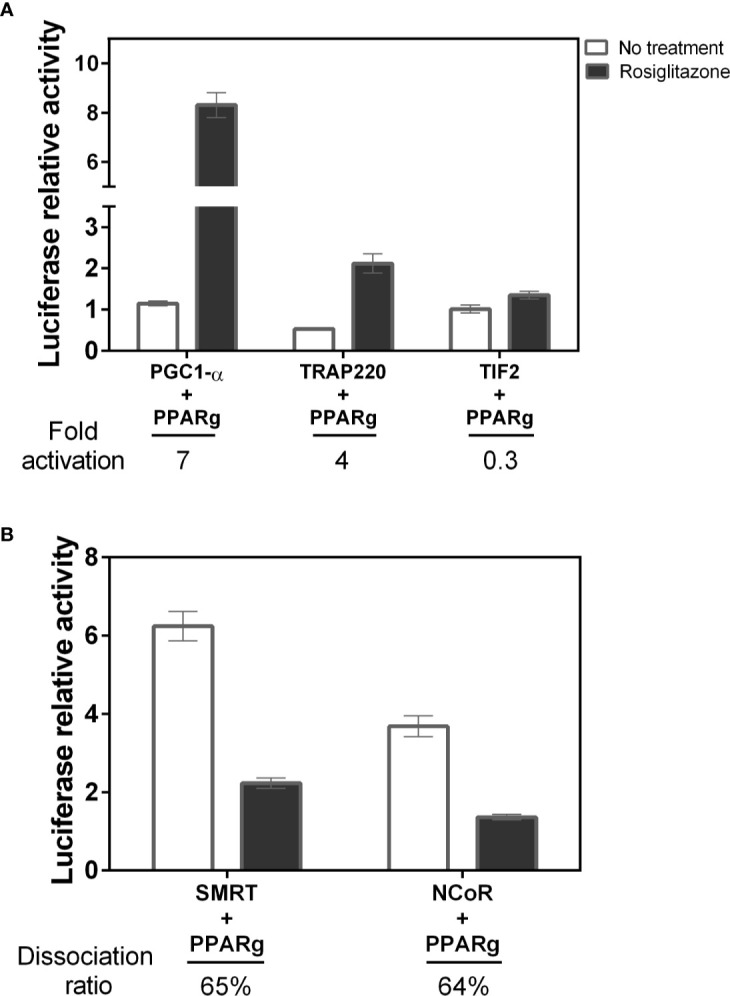
Affinity of different coregulators with PPARγ. **(A)** The interaction with the PGC1-α coactivator was the highest among the coactivators studied, followed by TRAP220 that maintains the high activation due to the ligand. The TIF2 coactivator did not have a large increase in the presence of the ligand, suggesting low interaction after ligand binding. B) Among the corepressors the SMRT had a higher affinity than NCoR. Four assays in biological triplicate were performed to coactivators n=12, and three for corepressors CoRs n=9. **(B)** Among the corepressors the SMRT had higher affinity than NCoR Error bars, SEM. (n=9).

To evaluate if phosphorylation could increase coactivator and/or decrease corepressor interaction with PPARγ, we perform mammalian two-hybrid assays comparing PPARγ interaction with the selected coregulators (PGC1-α, TRAP220, TIF2, NCoR and SMRT). Firstly, we measured the PPARγ binding preferences with the chosen coregulators (**Figure 2**). The results show that within the coactivators, PGC1-α had the highest interaction with PPARγ (7-fold), followed by TRAP220 (4-fold). Interestingly, our construct of TIF2 did not presented significant changes in its interaction due to ligand responsiveness, suggesting low PPARγ binding due to agonist effect. Among the corepressors, both showed a similar dissociation rate, in the presence of the ligand, of 65% and 64% respectively for SMRT and NCoR. In addition, the initial interaction rate (No treatment) of SMRT is higher, suggesting a preferential binding to PPARγ.

**Figure 3:**
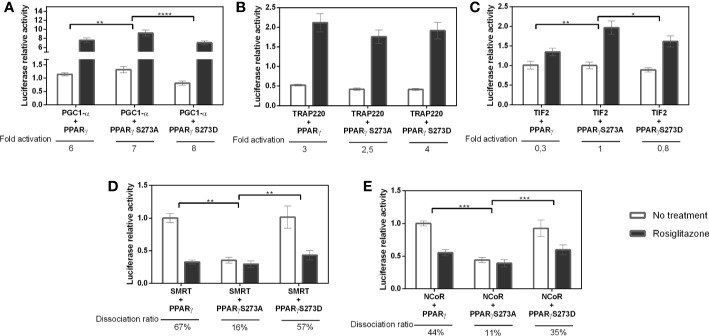
Interaction between PPARγ and coregulators in different phosphorylation states. Interaction measured by mammalian two-hybrid assays were performed in COS-7 cells for **(A)** PGC1-α, **(B)** TRAP-220, and **(C)** TIF-2 coactivators and 239T cells for **(D)** SMRT and **(E)** NCoR corepressors. Error bars, SEM. (n=15) Statistical analysis: two-way ANOVA. P values: p ≤ 0.05* p ≤ 0.01** p ≤ 0.001***; p ≤ 0.0001****. The coactivators PGC1-α and TIF2 presented increased interaction with PPARγS273A mutant, while the corepressors presented decreased interaction with the same receptor mutant.

Furthermore, we measured the coregulators interaction with PPARγ in various phosphorylation states. Despite having different interaction rates, TIF2 and PGC1-α coactivators (**Figures 3A, C**), presented similar interaction profile with the PPARγ wt, PPARγ S273A, and PPARγ S273D, both presenting higher interaction with phosphorylation-defective mutant (S273A). Moreover, the interaction with the phosphomimic mutant (S273D) presented similar behavior to the wt receptor, indicating that these coactivators binding are sensitive to S273 phosphorylation and suggesting an increased binding of these coactivators in absence of PPARγ phosphorylation. This interaction profile agrees with the activation profile seen in **Figure 1**, confirming that the lack of phosphorylation might increase coactivators binding. Nevertheless, the TRAP220 did not show interaction changes with the receptor in any phosphorylation state, suggesting that its binding to the receptor occurs independently of the PPARγ phosphorylation state (**Figure 3B**).

Regarding the corepressors, both were influenced by PPARγ dephosphorylation, as they presented the lowest interaction with S273A mutant (**Figures 3D, E**), and a decrease in dissociation ratio after Rosiglitazone addition. In contrast, the PPARγ wt and the S273D mutant presented similar interaction activity with corepressors, for both SMRT (**Figure 3D**) and NCoR (**Figure 3E**), opposite behavior observed for the coactivator’s recruitment. Combined, these results confirm that the phosphorylation inhibition reduces the recruitment of NCoR and SMRT and, at the same time, increases the recruitment of PGC1-α and TIF2.

**Figure 4:**
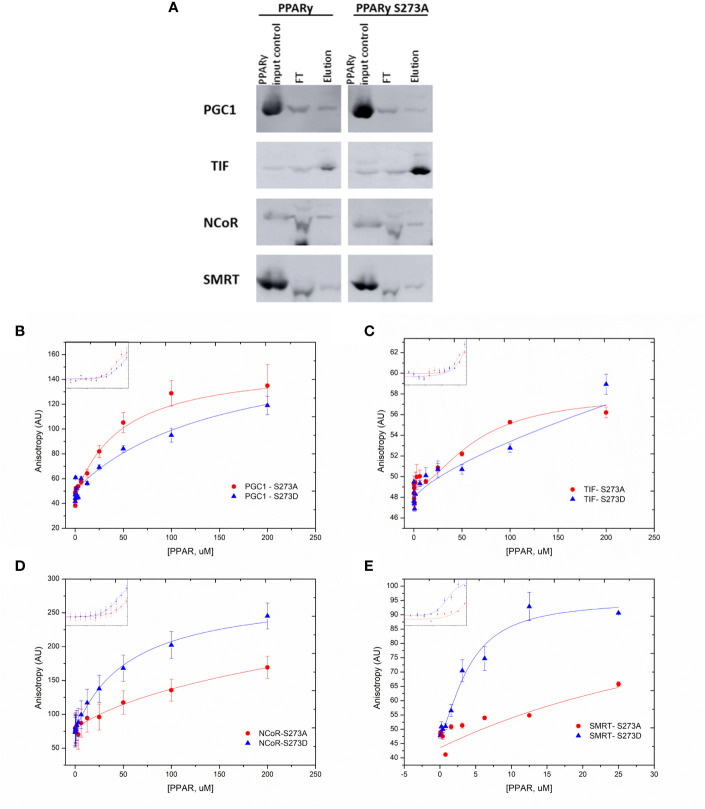
Differential PPARg: coregulator interactions. **(A)** Western blotting analysis of PPARg: coregulator complexes. Tagged coregulators protein extracts were used as bait to bind PPARγ and PPARγS273A by affinity chromatography. The confirmation of complex formation is showed using an antibody against PPARγ in pull-down eluted samples. **(B–E)** Fluorescence anisotropy measurements obtained from the titration of PPARγ wt, S273A and S273D mutants into fluorescein-labeled coregulators. **(B)** PGC1-α anisotropy measurements. **(C)** TIF2 anisotropy measurements. **(D)** NCoR anisotropy measurements. **(E)** SMRT anisotropy measurements. The experimental controls and kd values are in **Supplementary Material**.

In addition, these PPARγ:coregulator interactions were confirmed by pull-down assays (**Figure 4A**). We used tagged coregulators protein, as the bait to purify excess of PPARγ and of PPARγS273A by affinity chromatography, generating PPARγ:coregulator complexes. Although very useful to confirm the existence of these complexes, this assay did not provide enough accuracy to quantify the differences in affinities between the four coregulators chosen and the different PPARγ phosphorylation states. However, qualitatively, it is possible to observe that PPARγ binds to all the coregulators in this assay, but the expression of these coregulators in *E. coli* system is variable in terms of protein content and different affinity comparisons are not possible to perform.

To confirm these differential interactions, we perform a fluorescence anisotropy assay within the coregulators that were responsive to S273 phosphorylation (**Figures 4B–E**). In this assay, coregulators were expressed in *E. coli*, purified by affinity column and labeled with FITC. Our results show that PGC1-α binds better to the S273A mutant (**Figure 4B**) (Kd = 46.9 ± 10) in comparison to S273D mutant (Kd = 153.5 ± 44.4, respectively), confirming our previous results (**Figure 3A**). TIF2 presented very low affinities to binding to all the PPARs, which is reflected by the low amplitude of the anisotropy binding curve and by Kds not determined because curves did not achieved saturation (**Figure 4C**), This result confirms that shown in two hybrid assays (**Figure 2A**). Besides this, a preference for the S273 mutant is suggested due to the binding curve shape. Both corepressors presented better affinities with phosphomimic mutant S273D (**Figures 4D, E**), and, as also shown in two hybrid assays, SMRT presented better affinity in comparison to PGC-1 (Kd = 4.06 ± 1.01 uM, and Kd = 55.8 ± 2.9 uM). Together, these data demonstrate strong binding preferences among PPAR mutants, which confirms our two-hybrid assays (**Figure 3**) results. It is important to mention that this is the first time that bigger constructions of coregulators were assayed in this kind of fluorescence assays, while the most common data about this kind of interaction is presented in the literature using the ID peptides of these molecules. Despite that, the Kds may not be compared to the found ones.

### The Phosphorylation State Alters Adipogenesis Profile but Not Necessarily Coregulators Gene Expression

**Figure 5:**
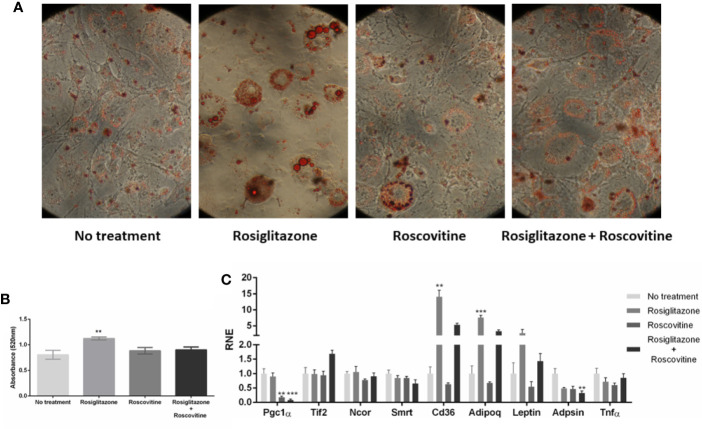
Adipocyte differentiation in different states of PPARγ phosphprylation. **(A)** Images of the 8th day of treatment for differentiation into adipocytes. 3T3-L1 cells after 7 days of treatment with differentiation cocktail stained with Oil Red O in 40× lens. During the differentiation process, were added1 μM of Rosiglitazone, 10 μM of Roscovitine, or both treatments. **(B)** Absorbance measurement of differentiated cells into adipocytes. After each treatment, the cells were stained with Oil Red O and the absorbance was measured by a spectrophotometer. Statistical differences were measured by one-way ANOVA, comparing the different treatments, values of p < 0.05/** 0.001/*** 0.001. The treatment with Rosiglitazone showed greater absorbance, therefore a higher level of differentiation. **(C)** Gene expression of genes of the studied coregulators, and some of the PPARγ regulated genes that were reported be dysregulated by S273 phosphorylated state (Cd36, Adipoq, Leptin, Adpsin, and Tnf-α). The statistical analysis was performed by Kruskal-Wallis test (non-parametric), followed by the Dunn *post hoc* test comparing the untreated condition with each one of treated conditions. P values: p ≤ 0.01**; p ≤ 0.001***.

To investigate whether the differential coupling of coregulators is due to differential protein recruitment or to changes in coregulators gene expression, we performed gene expression analysis on differentiated 3T3L1 cells (**Figure 5**). The cells were treated with Rosiglitazone, PPARγ agonist, known for increasing its adipogenic capacity (43) and for PPARγ phosphorylation inhibition (20); with Roscovitine, a CKD5 inhibitor which has already been shown to significantly suppress CDK5-mediated phosphorylation, improving the expression of most of the genes regulated by PPARγ S273 phosphorylation (44, 45); and by both ligands. In this assay, the two compounds were used as a treatment during adipogenesis to evaluate whether CDK5 inhibition phosphorylation capacity would modify the expression profile, of the chosen coactivators and the adipogenic capacity of PPARγ.

First, we observed that adipogenesis were reduced in Roscovitine and Roscovitine+Rosiglitazone treatments, as it is shown in **Figures 5A, B**. Only Rosiglitazone effectively induced adipocyte differentiation, which is evidenced by the size of the lipid droplets coloured by Oil Red O, and by the Oil Red O absorbance measurements, suggesting that Roscovitine impairs white adipocyte (WAT) differentiation. As it was reported, Roscovitine can induce browning of adipose cells, turning the characteristic bigger lipid droplet in WAT in smaller and multiple lipid droplets that are usual in brown adipose tissue (BAT) (45).

The gene expression results confirm that the differences in PPARγ:coregulators interaction were not due differential availability of coregulators in different PPARγ phosphorylation state. Therefore, it confirms the hypothesis of differential interaction profiles that leads to differential activation. Among all the assayed coregulators, we observed a decreased expression of PGC1-α, while TIF-2, NCoR and SMRT kept the same expression rates in all the treatments. In another words, PGC1-α was the only coregulator downregulated by Roscovitine treatment, even when this compound was associated to Rosiglitazone. Interestingly, as it was previous shown, the PGC1-α is the PPARγ most recruited coactivator after Rosiglitazone treatment (**Figure 2A**), and, that this interaction increased in the absence of PPARγ phosphorylation (**Figure 3A**). However, CDK5 inhibition seems to decrease this gene expression, suggesting a fine regulation in this coactivator recruitment, which should be specific and strong enough to overpass the limiting expression rates of it.

Additionally, the other coregulators did not presented differences in gene expression rates in all the treatments, suggesting that, for TIF-2, NCoR and SMRT, changes in PPARγ binding, even in different PPARγ phosphorylation states, are probably caused by different interaction modes, and not due to increased or decreased availability of these proteins. Moreover, we also observed that the PPARγ regulated genes Cd36, Adipoq and Leptin were upregulated by rosiglitazone, while Adpsin was downregulated by Rosiglitazone + Roscovitine, and that TNF-α did not changed expression profile in all the treatments. These results suggest improved adipogenesis after agonist treatment (20, 43), phospho-protective effects against adipogenesis after Roscovitine treatment (45, 46), and no inflammation induced responses in all the conditions, as expected.

### IDs Preferences for PPARγ-Coregulator Interaction

**Figure 6:**
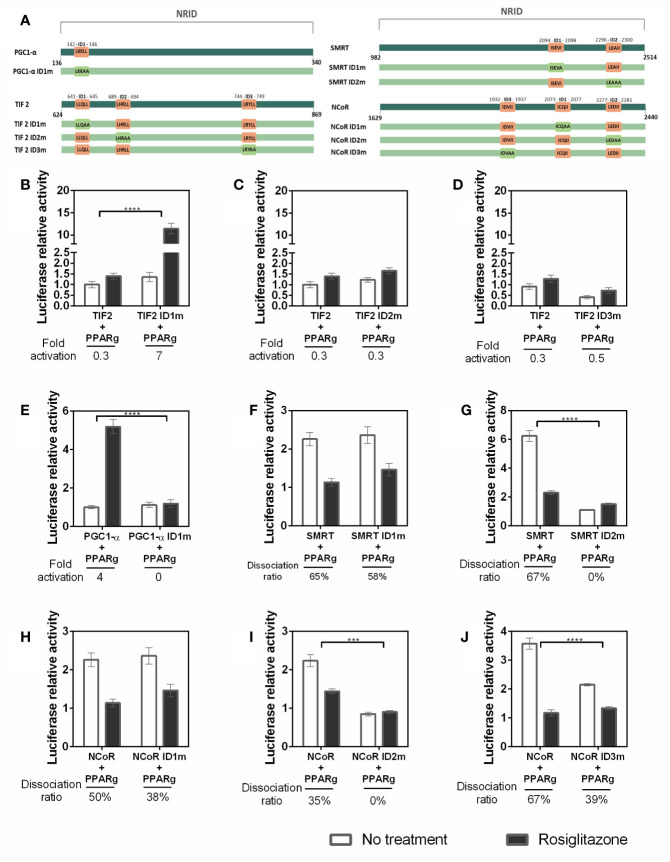
Affinity of the coregulators IDs with PPARγ. **(A)** Representative image of the IDs in the sequences used and their respective mutations. In dark green are the original sequences, in light green are the mutated sequence. The original IDs sequence are in orange squares and the mutations on IDs are presented on light green squares. **(B–J)** Mammalian two hybrid assays were performed to evaluate whether the mutation on each interaction domain (ID) of the coregulators alters the interaction with PPARγ. **(B)** Comparison between interaction with TIF2 coactivator wt and PPARγ, and the ID1 of TIF2 mutated (TIF2 ID1m) and PPARγ. **(C)** ID2 of TIF2 coactivator mutated. **(D)** ID3 of TIF2 coactivator mutated. **(E)** ID1 of PGC1-α coactivator mutated. **(F)** ID1 of SMRT corepressor mutated. **(G)** ID2 of SMRT corepressor mutated. **(H)** ID1 of NCoR corepressor mutated. **(I)** ID2 of the NCoR corepressor mutated. **(J)** ID3 of the NCoR corepressor mutated. Error bars, SEM. (n = 15) Statistical analysis: two-way ANOVA. p-value: P values: p ≤ 0.001***; p ≤ 0.0001****. For the TIF coactivator the withdrawal of ID1 improves the interaction, in this case we can say that ID1 interferes with the interaction PPARγ-TIF2 and ID3 of the same coactivator is the most important for the interaction. In the case of PGC1-α, we only have 1 ID and when it is not present the interaction is broken. For SMRT and NCoR, ID 2 is important, ID1 does not change the interaction and ID3 of NCoR seems to contribute to the interaction with PPARγ.

Additionally, to identify preferential binding of PPARγ to each coregulator ID, we performed mammalian two-hybrid assays with coregulators using wt and ID defective constructs of coregulators (**Figure 6A**), by mutating their active IDs. Hence, the coactivators IDs, which have the LXXLL motifs recognized as the ID, had their last two leucine replaced by alanine residues, resulting in the LXXAA motif. The corepressors domains had the IXX(V/I) motif modified by the substitution of isoleucine or valine residues for alanine, resulting in IXXAA motif.

Our searching for the preferential IDs for PPARγ wt - CoAs binding reveal a panel of ID binding preferences. Firstly, each TIF 2 ID contributes differently to the PPARγ interaction. The ID1 absence (**Figure 6B**) increased the interaction between TIF2 and PPARγ, indicating that its presence may be disrupting the binding of TIF2 to the PPARγ, possibly by competition between the IDs or unfavorable conformation of the coactivator structure when the ID1 is present (**Figures 6C, D**). The ID2 mutation (**Figure 6C**) does not altered the CoA-PPARγ binding, which means that this ID does not contribute for PPARγ-TIF2 interaction. However, the lack of ID3 (**Figure 6D**) drastically reduced the interaction with PPARγ, demonstrating that this ID possibly is the most important for PPARγ-TIF2 binding. Concerning PPARγ–PGC1-α binding, the mutation on the unique PGC1-α ID (**Figure 6E**) decreased the Rosiglitazone-induced interaction with PPARγ, as expected.

We also checked the preferential IDs in the PPARγ wt - CoR binding. Our results show that the lack of SMRT ID1 (**Figure 6F**) did not provoked any significant differences in the interaction with the receptor, as the efficiency of dissociation of this CoR in the presence of the ligand was also maintained. However, mutation of SMRT ID2 (**Figure 6G**) reduced the PPARγ-SMRT binding about 6-fold in comparison with SMRT wt, showing that this ID possibly is the most important in the PPARγ-SMRT interaction. For NCoR, the lack of ID1(**Figure 6H**) also did not significantly change its interaction with PPARγ, as it was observed for SMRT, but the NCoR ID2 absence (**Figure 6I**) abolished the PPARγ-NCoR interaction, pointing to the importance of this ID in the corepressor-receptor interaction, as it was also seen for SMRT. Finally, the absence of NCoR ID3 (**Figure 6J**) decreases the PPARγ-NCoR interaction, but the reduction found was lower than the found for ID2, suggesting that both ID2 and ID3 contributes in the PPARγ-NCoR interaction, but ID2 is likely the most important one.

### The IDs Preferences for PPARγ Binding Change Due the Phosphorylation State

**Figure 7:**
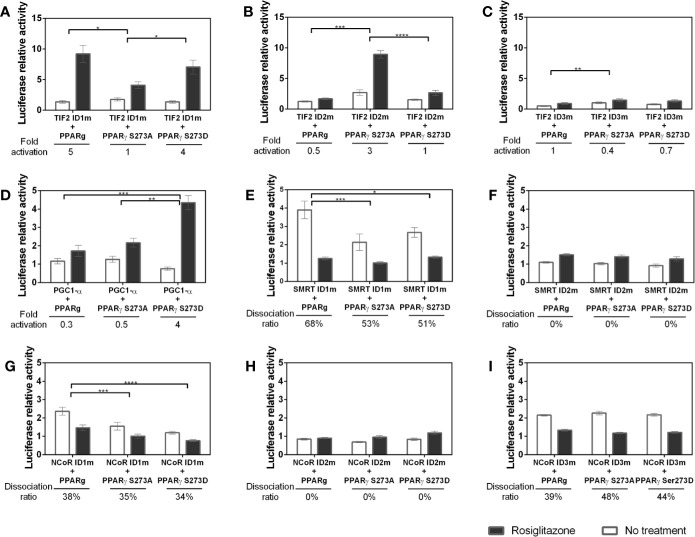
Interaction between coregulators and PPARγ in different phosphorylation states. Mammalian two hybrid assays were performed to evaluate whether the S273 mutation in the receptor interferes with its interaction with the coregulators. The PPARγ S273A mutant prevents the occurrence of phosphorylation and the PPARγ S273D mutant is a phosphomimic. **(A–C)** Interaction between TIF2 mutants and PPARγ in different phosphorylation states. **(D)** Interaction between PGC1-α mutant and PPARγ in different phosphorylation states. **(E, F)** Interaction between SMRT mutants and PPARγ in different phosphorylation states. **(G–I)** Interacton between NCoR mutants and PPARγ in different phosphorylation states. Error bars, SEM. (n = 15) Statistical analysis: two-way ANOVA. P values: p ≤ 0.05*; p ≤ 0.01**; p ≤ 0.001***; p ≤ 0.0001****.

To evaluate whether the PPARγ S273 phosphorylation state modifies the PPARγ-coregulators interaction profile we also performed the mammalian two hybrid assays with the PPARγ S273 mutants and coregulators with IDs mutants. Our results show that changes in TIF2 IDs (**Figures 7A–C**) presented considerable variation in the interaction with the different PPARγ phosphorylation states. The absence of ID1 (**Figure 7A**) increased the responsiveness of PPARγ wt to the Rosiglitazone ligand (as it was shown in **Figure 6B** and in the first bar of **Figure 7A**). However, when the phosphorylation is inhibited (PPARγ S273A) the PPARγ-TIF2 interaction decreased, and, in the phosphorylation-mimicking condition (PPARγ S273D) no significant differences between PPARγ wt was observed. Inversely, the absence of ID2 (**Figure 7B**) increased the interaction of TIF2 with the receptor when the phosphorylation is inhibited (PPARγ S273A) and decreased this interaction with the PPARγ wt and in the phosphorylation mimetic receptor (PPARγ S273D). Mutation on ID3 of TIF2 dramatically decreased receptor interaction under all conditions (**Figure 7C**). Together, these indicate that the TIF2 ID3 is the most important for the PPARγ interaction, and IDs 1 and 2 are affected by S273 phosphorylation. ID1 may be important to help in the protein-protein interaction for non-phosphorylated PPARγ, and the ID2 may be important for the phosphorylated PPARγ interactions.

The lack of ID1 in PGC1-α (**Figure 7D**) show similar interaction of this CoA with PPARγ wt and PPARγ S273A. However, the phosphorylation (PPARγ S273D) substantially increased the interaction with PPARγ in the presence of ligand, unveiling that this coactivator may bind to an additional region of the receptor uniquely when it is phosphorylated.

The mutation of SMRT ID1 presented decreased interaction with both conditions of PPARγ mutants (**Figure 7E**). This suggests that the structural changes provoked by S273 affect the interaction with this ID. The ID2 mutation (**Figure 7F**) decreased the interaction between PPARγ and SMRT in all states of phosphorylation. This profile was already observed in **Figure 6G** and are consistent with other studies that demonstrate that this is the most important ID for receptor interaction (47, 48). Moreover, no significant difference was observed between the mutation of this ID and PPARγ phosphorylation.

NCoR ID1 mutation (**Figure 7G**) was also able to reduce the interaction with both mutants, S273A and S273D. Mutation on ID2 (**Figure 7H**), as the SMRT ID2m, presented the lower interaction with PPARγ in all conditions. The result shows that there is a reduction in the interaction between NCoR with inactive ID2 independent of the state of receptor phosphorylation, but due to the PPARγ preference for binding *via* this ID. The ID3 mutation (**Figure 7I**) showed no difference due the phosphorylation state, which indicates that this ID is irrelevant in the interaction corepressor-receptor due to phosphorylation/dephosphorylation of PPARγ.

### CDK5 Modifies PPARγ-Coregulator Interaction

Finally, to evaluating the preferential coregulators IDs for PPARγ binding and the changes in this preference caused by receptor’s phosphorylation state, we performed some assays in the presence of CDK5, to check if this enzyme would modify the interaction profile with the different coregulators. These assays allow us to estimate what occurs in the cell at the beginning of phosphorylation, while in the previous assays, using S273 mutants, we evaluate the result of phosphorylation in the PPARγ-coregulators binding.

**Figure 8:**
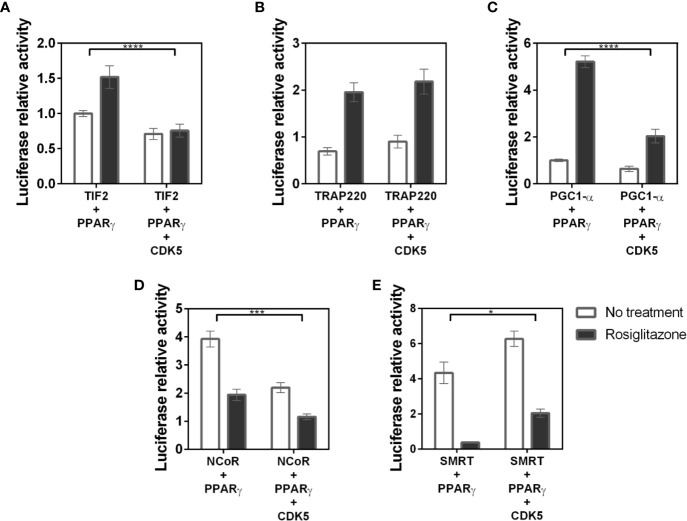
Interaction between coregulators and the PPARγ receptor in the presence of the CDK5 enzyme. Mammalian two hybrid assays to evaluate if the presence of the CDK5 enzyme, responsible for the phosphorylation of S273 in the receptor, interferes with PPARγ:coregulators interaction. **(A)** Interaction of the TIF 2 coactivator with PPARγ in the absence and presence of CDK5. **(B)** Interaction of the TRAP220 coactivator with PPARγ in the absence and presence of CDK5. **(C)** Interaction of the PGC1-α coactivator with PPARγ in the absence and presence of CDK5. **(D)** Interaction of the NCoR corepressor with PPARγ in the absence and presence of CDK5. **(E)** Interaction of the SMRT corepressor with PPARγ in the absence and presence of CDK5. Error bars, SEM. (n = 15) Statistical analysis: two-way ANOVA. P values: p ≤ 0.05*; p ≤ 0.001***; p ≤ 0.0001****. PGC1-α, TIF2 and NCoR showed dissociation of the receptor in the presence of CDK5 while SMRT increased the association with the receptor.

Our results show that PGC1-α, TIF2, and NCoR assays (**Figures 8A, C, D**, respectively) decreased receptor interaction in presence of CDK5. The PGC1-α-PPARγ decreased from 5-fold in absence of CDK5 to 2-fold. TIF2 decreased PPARγ binding from 1.5-fold to 0.7-fold, indicating that the interaction with the receptor was missed, and NCoR interaction decreases from 4 to 2-fold. Meanwhile, the SMRT corepressor (**Figure 8E**) displayed opposite behavior, increasing interaction with PPARγ in the presence of CDK5, indicating that the enzyme may play some roles as PPARγ-corepressor coupling, as previously suggested (31). Interestingly, for TRAP220, the CDK5 presence did not change the PPARγ-coactivator interaction, as it was shown for the PPARγ mutants. All these results allow us to infer that the enzyme may alter the interaction profile by competing or coupling coregulators to the PPARγ binding site, depending on the coregulator, and that TRAP is not affected by PPARγ phosphorylation.

**Figure 9:**
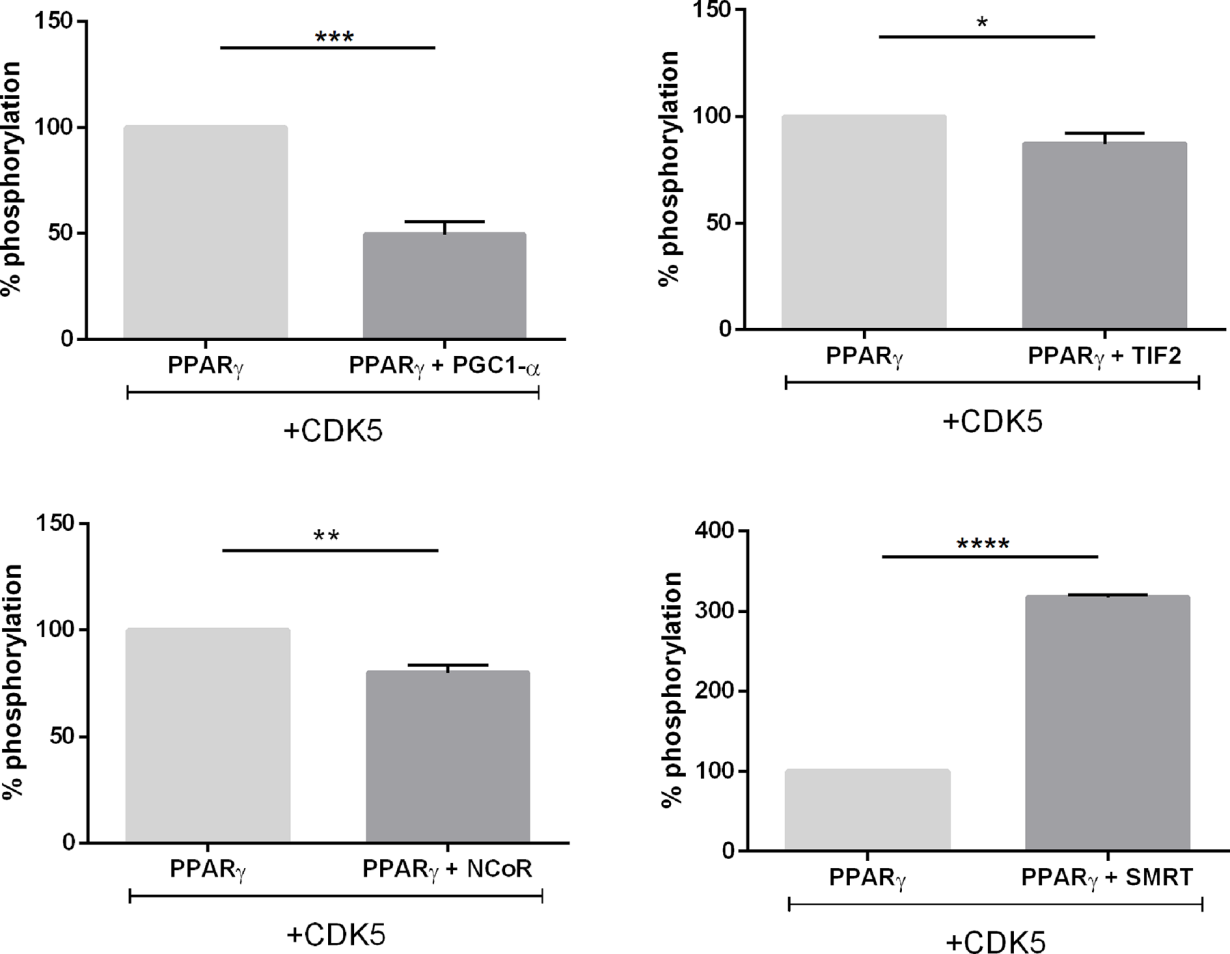
In vitro phosphorylation assay. Luminescence signal produced as consequence of the ADP production *in vitro* reaction containing CDK5/p35 kinase, ATP and PPARγ, and the complexes with coregulators. All the luminescence signals were normalized by PPARγ condition which is 100% of phosphorylation. Error bars, SEM, (n = 3). Statistical analysis: one-way ANOVA. P values: p ≤ 0.05*; p ≤ 0.01**; p ≤ 0.001***; p ≤ 0.0001****. The complex PPARγ + SMRT presented increased luminescence while the other three complexes presented decreased luminescence.

Still to confirm that CDK5 presence disturbs the PPARγ interaction with coregulators we perform *in vitro* phosphorylation assay with heterologous expressed PPARγ, and PPARγ-coregulators complexes formed in the pull-down assays (**Figure 9**). The phosphorylation of PPARγ by CDK5 was used as the control, set up as 100% of phosphorylation, and the increase or decrease of the PPARγ phosphorylation due to coregulator presence was compared with this condition. Our results show that PPARγ:SMRT complex presented an increase of 164% in phosphorylation rate, confirming our cellular assays (**Figure 8E**) that showed that CDK5 presence increases SMRT interaction with PPARγ. Moreover, as also shown in our cellular assays, the other three complexes presented reduced interaction in CDK5 presence, been PPARγ:PGC1-α complex the one which presented the major interaction disruption, decreasing 52% when added CDK5 in the system. In addition, TIF2 presented the lower interaction difference (11%), possibly due to its weak interaction with PPARγ even in absence of CDK5. PPARγ:NCoR complex presented a 17% of reduction of phosphorylation rate, indicating that NCoR may compete with CDK5-PPARγ for docking.

## Discussion

Previous studies have reported that Ser273 phosphorylation of PPARγ LBD is related to obesity-induced development of insulin resistance (14, 20, 21). A key question to understand the mechanisms of action of this pathway is to elucidate how this phosphorylation influences the PPARγ activation. Our results showed that both phosphorylation status and CDK5 presence can indeed alter the PPARγ activation (**Figure 1**). Moreover, our results show that these differences on activation are due the differential interaction with coregulator proteins (**Figures 2**–**4**).

As it is well known, the formation of protein-protein complexes and subsequent transcriptional regulation is completely dependent on the structure (49, 50). PTM-dependent interactions occur through structural changes that creates binding sites for a range of IDs (51). Our results showed the PPARγ binding to coregulators occurs and presented different preferences of binding (**Figures 3** and **4**), that may be modified by phosphorylation. Additionally, our results show that these binding preferences dependent of PPARγ phosphorylation state is not due to differential expression of the coregulators or guided by increased availability of a determined coregulator when phosphorylation is suppressed (**Figure 5**). On the contrary, the decreased expression of PGC1-α when phosphorylation is inhibited did not change the higher preference of the receptor for this coactivator (52, 53).

Through cellular assays, we demonstrate that the coactivator TRAP220 was not responsive to Ser 273 phosphorylation nor to the presence of the CDK5 enzyme (**Figures 3** and **6**). One possible explanation for this lack of responsiveness is that, although it has 3 different IDs, this coactivator probably binds to PPARγ only by the canonical interface formed by PPARγ H12 relocation and H3, H4 and H5, without any other additional interaction. Thus, neither phosphorylation, nor CDK5 presence affect the opposite face of the receptor, not affecting the receptor-coactivator interaction. However, both TIF2 and PGC1-α coactivators exhibited a different behavior, presenting higher interaction with PPARγ in the phosphorylation inhibited state (**Figure 3**).

Additionally, PGC1-α, which is known as PPARγ’s preferred coactivator (52, 53), showed preferential binding to PPARγ wt by its unique ID (**Figure 6E**). Moreover, this coactivator shows to make additional contacts with the receptor in the phosphorylated state, as the deletion of ID1 increased the interaction between the PGC1-α and PPARγ S273D (**Figure 7D**). Possibly, this contact may be mediated by an additional and inverted LXXLL motif that exists between amino acids 210 to 214 of PGC1-α, which has been shown to interact with other NRs, such as ERRα (54) and is called L3. Despite it is well known that the main PGC1-α ID with most NRs is the ID corresponding to L2 (aas 144-149, here called ID1), our results show that when the strongest ID is inactivated, other motifs, as L3 becomes to anchor to the PPARγ, but only if the S273 is phosphorylated. Nevertheless, the existence of this phosphorylation-responsive interaction might explain the decreased interaction of PGC1-α wt with the phosphomimic mutant PPARγ S273D (**Figure 3A**). In this case, phosphorylation would increase the affinity of L3 motif for the receptor, generating a competition between L2 (or ID1) and L3 motifs, which, for structural reasons, cannot bind at the same time to the receptor, weakening the interaction that was previously made only *via* ID1-H12. This possibly occurs through the CDK5-PGC1-α competition on the PPARγ coupling site. Interestingly, the decreased PGC1-α expression in adipose tissue when such phosphorylation occurs is associated with increased insulin resistance (55, 56).

Interestingly, TIF2, which did not present high preference to bind PPARγ (**Figure 2** and **4C**), was also responsive to phosphorylation. Its role in regulating adipose tissue homeostasis, and its expression appears to be linked to increased insulin resistance in mice (57). Our results show that it binds to PPARγ canonically *via* ID3 (**Figure 6D**), however its other IDs are responsive to phosphorylation in opposite manners. According to our data, while ID1 seems to bind better when the phosphorylation is inhibited (**Figure 7A**), ID2 seems to bind better to the phosphorylated receptor (**Figure 7B**). This exchange of interaction interfaces with the receptor due to its phosphorylation state might induces exposure of different interaction surfaces to factors in the transcription activation/repression complex and may lead to different metabolic responses. This type of modular protein IDs is used by the cells as a broad device to decode and respond to the state of its protein, with different IDs, being dedicated to the selective recognition of distinct PTMs (51).

Concerning corepressors and IDs interaction profile, NCoR and SMRT presented some similar behavior. Interestingly, our results showed that there are differences in the IDs recruitment depending on the corepressor. This difference may be explained due to the different mechanisms of binding of the ID1, ID2 and ID3 to the receptor, related to the variants on IDs motifs which are LXXXIXX (V/I) IXXX (Y/F), LXXIIXXXL, and IXXIIXXXI, respectively (37, 58). Each of them has its own particularities on receptor binding. The ID2 for example, attach to PPARα by adopting an irregular three turn helix that fits tightly into a receptor groove formed by open conformation of H12. In this case, this surface can also act as a coactivator binding site (59). Both corepressors showed strongest interaction with PPARγ *via* ID2, corroborating with previous studies that demonstrate the importance of this ID to PPARγ interaction (48). On the other side, both ID1 seems to have little or no interaction with PPARγ. However, NCoR ID3 appears to be responsive to phosphorylation, as the lack of ID1 decreased the PPARγ binding in phosphorylated and no phosphorylated state, and the absence of ID3 did not respond to phosphorylation (**Figure 7**). This NCoR ID3 response to phosphorylation suggests that possible alternative contacts might be formed between this NCoR ID and the S273 region, as the S replacement for A or D amino acids might provoke particular conformational modifications in PPARγ structure. Interestingly, although the used isoform of SMRT does not have the ID3, the same responsiveness to the phosphorylation was observed, since the lack of ID1 also decreased PPARγ interaction when S273 is mutated.

Furthermore, our results revealed that the CDK5 presence also disturbs the PPARγ-coregulators interaction in different ways. Possibly the CDK5 has some coupling interface with PPARγ that overlaps the interaction interface with the coregulators, as it seems to compete with TIF2, PGC1-α, and NCoR (**Figure 8**). However, the interaction of PPARγ with SMRT is increased in the presence of CDK5, suggesting that, in this case, it is somehow coupling this corepressor, also through interaction interface intersection. These results were confirmed by *in vitro* phosphorylation assays were the complexes TIF2:PPARγ, PGC1-α:PPARγ, and NCoR:PPARγ presented increased ADP activity and SMRT:PPARγ presented the opposite profile (**Figure 9**).

This study adds details to the mechanisms of obesity induced by PPARγ phosphorylation. Our data not only confirm that the coregulators’ interaction profile could change due this phosphorylation (30, 31), but also show that this PTM could lead to new interactions sites within coregulators:PPARγ and coregulators:CDK5. A better understanding of this mechanism of action opens new pathways for anti-diabetic drug development. Previous studies show that there is a range of molecules that can bind to PPARγ preventing Ser273 phosphorylation, without cause the high activation characteristic of strong agonists (20–22, 27) and these results opened a new target possibility, the PPARγ:coregulator interaction. Inhibitors of this interaction can act either by binding to the binding groove formed by the IDs or by binding to the receptor’s H12 (60). Moreover, our results showed that in addition to these interaction sites, other unusual regions may have their interaction induced by the PPARγ phosphorylation state, further opening the range of possibilities for the new molecules searching.

**Figure 10:**
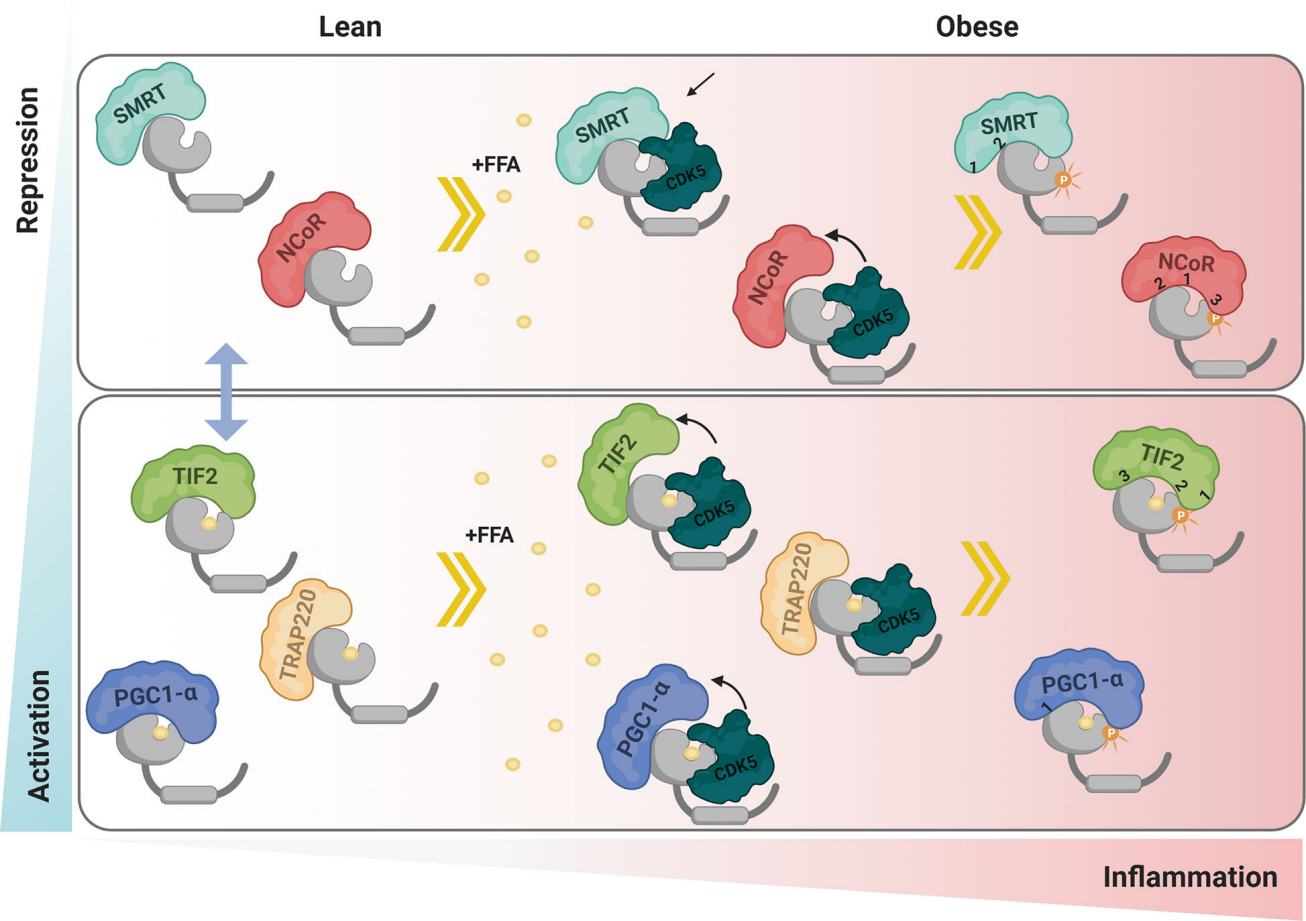
Proposed interaction mechanism. In lean adipose tissue the mechanism of interaction with coactivators and corepressors is in equilibrium, represented by the blue arrow. Under conditions of obesity, free fatty acids and other inflammatory factors act by activating the enzyme CDK5 that phosphorylates PPARγ. The presence of CDK5 generates an imbalance in the coregulators homeostasis, increasing the interaction of PPARγ with SMRT while decreasing with NCoR, PGC1-α and TIF2. Ser273 phosphorylation performed by CDK5 also modulates the interaction with coregulators. Both corepressors canonically bind *via* ID2-H12, and respond to modification in Ser273, both in the absence and presence of phosphorylation. PGC1-α, although interacting more strongly with the receptor *via* ID1, showed to make additional contact in a region near Ser273 that is favored in the presence of the ligand. TIF2 binds to H12 *via* ID3, however ID2 seems to interact better in the absence of phosphorylation and ID1 seems to interact better in the phosphorylation condition. TRAP 220 does not make contact near Ser 273, so it was not responsive to either phosphorylation or the presence of CDK5. Red represents the intensity of inflammation in adipose tissue. Blue represents levels of PPARγ activation due to interaction with the coregulators. The numbers 1, 2, and 3 represents the IDs (Created with BioRender.com).

Based on our results, we build a panel of possible PPARγ:coregulators interactions in different phosphorylation states (**Figure 10**). In summary, we showed that the phosphorylation inhibition increases PPARγ activation through higher interaction with PGC1-α and TIF2 coactivators and decreased interaction with SMRT and NCoR corepressors. The coregulators mutation assays results provide us insights to elucidate the importance of phosphorylation for the different coregulators anchorages possibilities. In particular our results show that the PGC1-α has been shown to make additional non-ID mediated contact with PPARγ in the region near Ser273. The ID3 of TIF2 coactivator seems to be the most important for canonical binding *via* H12 and IDs 1 and 2 make some contacts in the region near Ser273, depending on the phosphorylation state. Both tested corepressors showed that ID2 is the most important for the canonical interaction with PPARγ. However, ID1 is important in cases where modification of receptor S273 occurs, regardless of the receptor phosphorylation state. Finally, we have shown that the presence of CDK5 disrupts interaction with PGC-α, TIF2, and NCoR, probably through competition for the coupling site. Meantime, the interaction with SMRT is increased in this condition. These two different profiles of interaction indicate that the presence of CDK5 imbalance the coregulators natural activity.

## Data Availability Statement

The raw data supporting the conclusions of this article will be made available by the authors, without undue reservation.

## Author Contributions

AF designed the research and article and revised the article. MD, TT, FB, HR, FT, AO and LS performed the research. AM provided essential material, discussed results and methodology. MD and AF wrote the article. All authors contributed to the article and approved the submitted version.

## Funding

This work was supported by the “Fundação de Amparo à Pesquisa do Estado de São Paulo” (FAPESP) (grant #2016/22246-0, #2019/14465-1, and #2016/13480-9); “Conselho Nacional de Desenvolvimento Científico e Tecnológico” (CNPq) (#420416/2016-1); “Coordenação de Aperfeiçoamento de Pessoal de Nível Superior” (CAPES) (grant #88882.329749/2019-01 and #8887.373113/2019-00), and CNPEM.

## Conflict of Interest

The authors declare that the research was conducted in the absence of any commercial or financial relationships that could be construed as a potential conflict of interest.
