# Supplementary material for: PPARγ S273 Phosphorylation Modifies the Dynamics of Coregulator Proteins Recruitment
Source: Front Endocrinol (Lausanne). 2020 Nov 27;11:561256. doi: 10.3389/fendo.2020.561256 (PMC7729135; doi:10.3389/fendo.2020.561256)
Supplement: Supplementary file 1 [file DataSheet_1.pdf]

frontiers

PPARγ S273 phosphorylation modifies the dynamics of coregulator proteins recruitment.

Marieli Mariano Gonçalves Dias [1,2], Fernanda Aparecida Heleno Batista [1], Thais Helena Tittanegro[1], André Gustavo de Oliveira [4, 5], Albane Le Maire [1,3], Felipe Rafael Torres[1], Helder Veras Ribeiro Filho[1], Leonardo Reis Silveira [4, 5], and Ana Carolina Migliorini Figueira [1,2]

[1] Brazilian Biosciences National Laboratory (LNBio), Brazilian Center for Research in Energy and Materials (CNPEM), Campinas, SP, Brazil

[2] Graduate Program in Functional and Molecular Biology, Institute of Biology, State University of Campinas (Unicamp), Campinas, SP, Brazil

[3] Centre de Biochimie Structurale CNRS, Université de Montpellier, Montpellier, France

[4] Obesity and Comorbidities Research Center (OCRC), Campinas, Brazil.

[5] Department of Structural and Functional Biology, Institute of Biology, University of Campinas (UNICAMP), Campinas, Brazil

*** Correspondence:**
Ana Carolina Migliorini Figueira
(ana.figueira@lnbio.cnpem.br)
Phone number: + 55 19 3512 -1100. Postal Address: Brazilian Biosciences National Laboratory (LNBio), Brazilian Center for Research in Energy and Materials (CNPEM), Zip Code 13083-970, Campinas, Sao Paulo, Brazil.

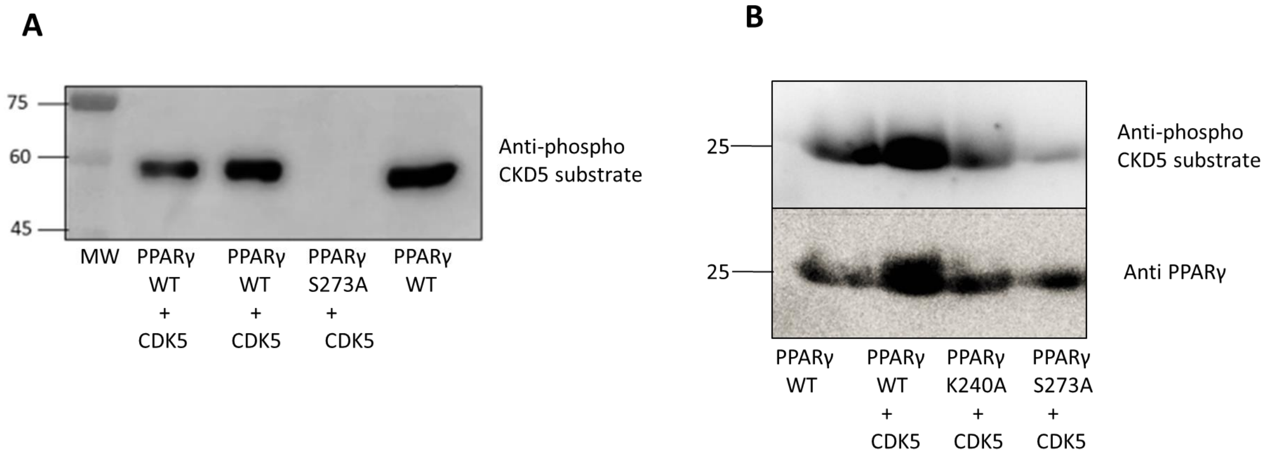

Supplementary Figure 1 – *Western blotting analysis of PPARγ phosphorylation state*. **A.** To analyze PPARγ phosphorylation status, we performed western blotting assay with transfected Flag-PPARγ and PPARγS273A in 293T cells in the presence of CDK5 and Flag-PPARγ without CDK5. After purification of cell extract by FLAG Tag Affinity Resin, proteins were electrophoresed on 12% polyacrylamide gel and transferred to the PVDF membrane by the semi-dry method and blocked with solution containing 3% BSA for 1 hour at room temperature or overnight at 4°C. Phosphorylated PPARγ were analyzed with anti Phospho-CDK Substrate Motif (Cell Signaling #9477), through Western Blotting after PPARγ immunoprecipitation through the Flag tag. After IP, 400ug of protein was loaded in each lane of the gel. 293T cells were transfected with PPARγ-Flag and Cdk5/p35 in order to confirm phosphorylation of the receptor when Cdk5 is superexpressed and to confirm the absence of phosphorylation, on S373A mutant. The anti-Cdk5 substrate antibody is responsible for the recognition of phosphorylated serine on the receptor. Loading of samples was checked by Ponceau (data not shown). **B.** The PPARγ phosphorylation status was also checked using heterologous expressed PPARγ LBD (30kDa) wt and the K240A and S273A mutants that prevent phosphorylation. These proteins were phosphorylated after incubation with CDK5/p35 complex. In this assay, 15uM of each PPARγ was incubated with 25 ng of purified CDK5/p35, at room temperature for 15 min, in the kinase assay reaction buffer (200mM Tris-HCl, pH 7.4, 100mM MgCl2 and 0.5 mg/ml BSA, SignalChem kinase assay buffer III) added 10 μM of ATP, in 12,5 μL of reaction volume. After that, proteins were electrophoresed on 12% polyacrylamide gel and transferred to the PVDF membrane by the semi-dry method and blocked with solution containing 3% BSA for 1 hour at room temperature or overnight at 4°C. Phosphorylated PPARγ were analyzed with anti Phospho-CDK Substrate Motif (Cell Signaling #9477), the input control was PPARγ, through Western Blotting.

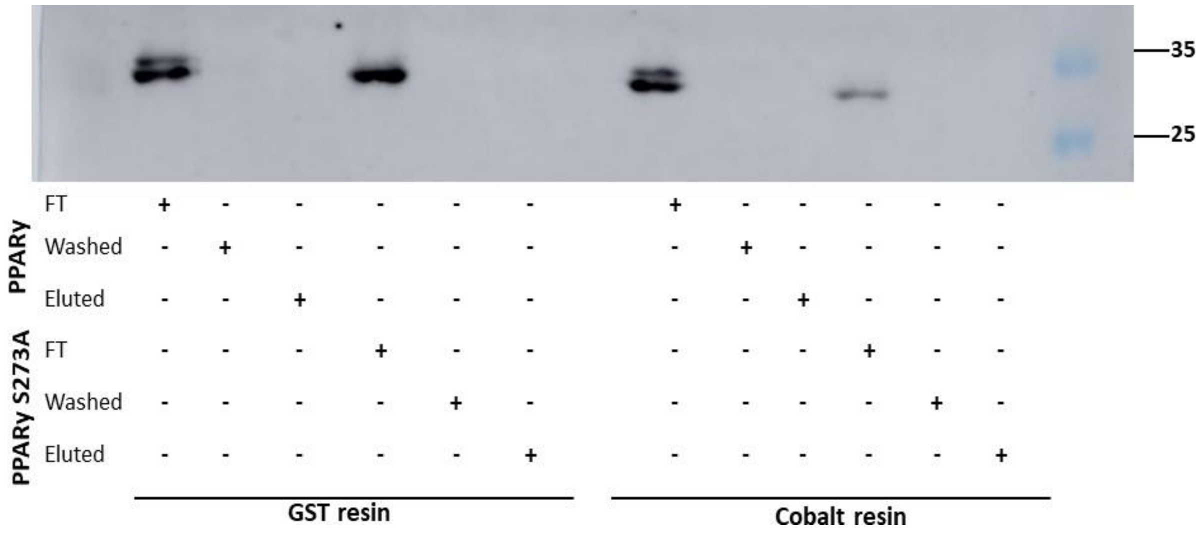

Supplementary Figure 2 – *Western blotting analysis of coregulators-PPARy pull-down.* Pull-down of purified PPARy and PPARy S273A used in pull-down assays as control of the experiment. The western blotting was performed using anti PPARy antibody.

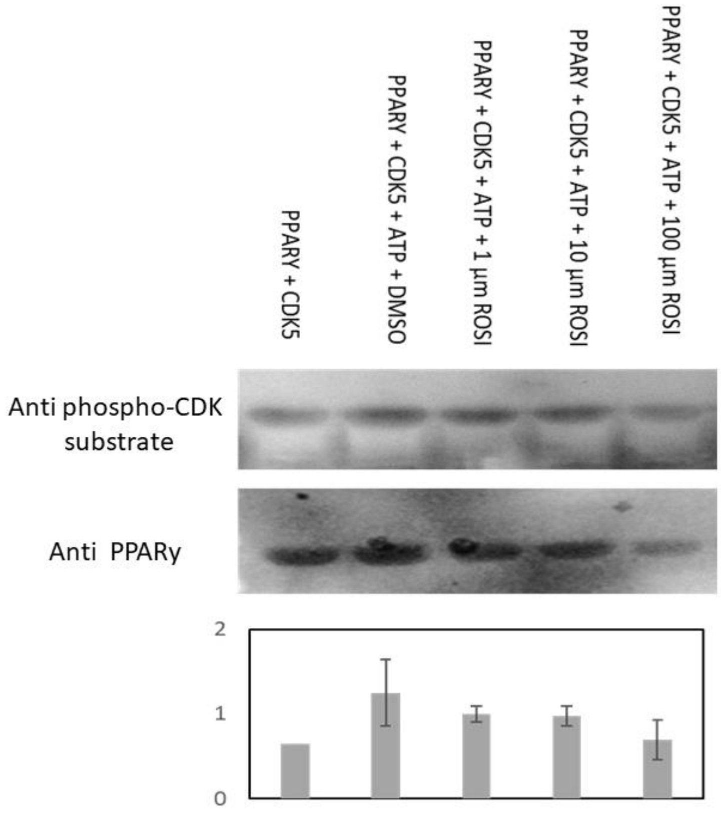

Supplementary Figure 3 – *Western blotting analysis of PPARy phosphorylation state.* To verify phosphorylation state of PPARy and possibly phosphorylation protection provoked by Rosiglitazone, the receptor was heterologous expressed, purified and incubated at 1uM with 1, 10 and 100uM Rosiglitazone. Western blotting assay was performed after proteins were electrophoresed on 12% polyacrylamide gel and transferred to the PVDF membrane by the semi-dry method and blocked with solution containing 3% BSA, for 1 hour, at room temperature or overnight at 4°C. Proteins were analyzed with anti Phospho-CDK Substrate Motif (Cell Signaling #9477) and with anti PPARy antibody. Here we present one representative image of 3 different experiments. The band densitometry reveals that Rosiglitazone at 1 and 10uM provoked little protection against phosphorylation, while 100uM Rosiglitazone caused protection of Ser 273, since the phosphorylation level is similar to the control (PPARy +CDK5 with no ATP – no phosphorylated condition).

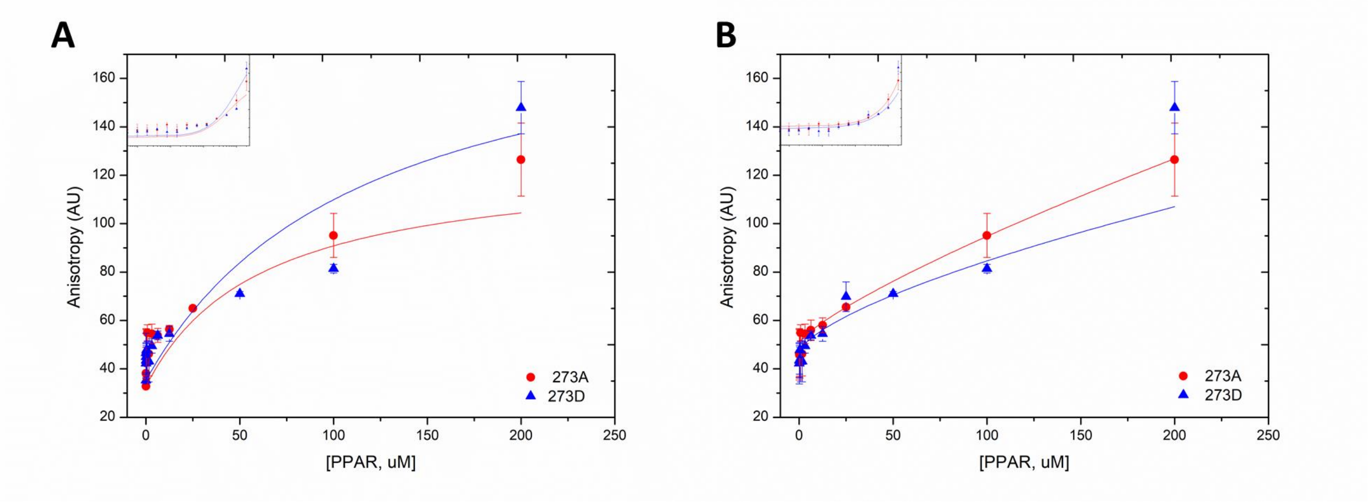

Supplementary figure 4 - *Experimental control of fluorescence anisotropy assay*. Non induced culture extracts were incubated in (A) GST and (B) Cobalt resins, labeled with FITC (50uL of FITC in 500uL of extract elution) and the affinity with PPARγ mutants were measured with serial dilutions of purified PPARγ S273A or S273D mutants (200μM to 6nM). The results show that some unspecific interactions between the extracts and all the PPARs happens only above 100uM, and Kd were impossible to obtain, indicating that the measured binding preferences found among all the PPARs mutant are specific.

Table 1 – Coregulators:PPARy interaction Kd (uM) values of fluorescence anisotropy assay. ND is non determined Kd.

	S273A (uM)		S273D (uM)	
protein	KD	sd	KD	sd
PGC-1	46.9	10	153.5	44.4
TIF	ND	ND	ND	ND
SMRT	ND	ND	4.06	1.01
NCOR	559.2	77	55.8	2.9

Table 2 – Sequence of primers used in qPCR gene expression analysis.

Gene Name	Forward primer	Reverse primer	product length (bp)
TATA box binding protein	CCAATGACTCCTATGACCCCTA	CAGCCAAGATTCACGGTAGAT	104
ribosomal protein L27	CTGGCCTTGCGCTTCAA	TCATGCCCACAAGGTACTCTGT	94
adiponectin	TGTTCCTCTTAATCCTGCCCA	CCAACCTGCACAAGTTCCCTT	104
leptin	GAGACCCCTGTGTCGGTTC	CTGCGTGTGTGAAATGTCATTG	139
complement factor D (cfd)	CATGCTCGGCCCTACATGG	CACAGAGTCGTCATCCGTCAC	129
tumor necrosis factor	CACTTGGTGGTTTGCTACG	CCAGACCCTCACACTCAGA	79
nuclear receptor coactivator 2 (Ncoa2)	AGCCATGAACTGTGTGCCAT	AATGTGTTGTGCAGAGCGTG	132
peroxisome proliferative activated receptor, coactivator 1 alpha (Ppargc1a)	TGAAGACGGATTGCCCTCAT	TGCTAAGACCGCTGCATTCA	191
nuclear receptor co-repressor 1	CAATCTGCCAGCAGTTACCA	CCCATGAGAGCCTTTCTGAT	111
nuclear receptor co-repressor 2 (Ncor2)	CATCTAGCCCACTCCTCCAG	GCGTGTAGTCCTGCGTAATG	102
CD36 molecule	GATCCGAACACAGCGTAGAT	GATCCGAACACAGCGTAGAT	123

Table 3 – Sequence of primers used to mutate corregulators, NCoR and SMRT ID1m and ID2m were kindly provided by Drª Albane Le Maire (Centre de Biochimie Structurale CNRS).

Primer	Sequence
PGC1a_ID1_F	GTCTCTACTTAAGAAGGCCGCACTGGCACCAG
PGC1a_ID1_R	GTGCCAGTGCGGCCTTCTTAAGTAGAGACGGC
TIF_ID1_F	CAAACTCCTGCAGGCGGCGACCACCAAATCTGA
TIF_ID1_R	AGATTTGGTGGTCGCCGCCTGCAGGAGTTTGGT
TIF_ID2_F	AATTTTGCACAGAGCCGCGCAGGACAGCAGT
TIF_ID2_R	GCTGTCCTGCGCGGCTCTGTGCAAAATTTTATG
TIF_ID3_F	GAGAATGCACTACTTCGCTATGCGGCAGATAAAGATGATAC
TIF_ID3_R	TATCATCTTTATCTGCCGCATAGCGAAGTAGTGCATTCTC
ID 3 NCoR_F	CAGCTAACTTCATAGACGT GGCCGCCACCCGGCAAATTGCCTCGG
ID 3 NCoR_R	CCGAGGCAATTTG CCGGGTGGCGGCCACGTCTATGAAGTTAGCTG
